# Potential biomarkers and therapeutic targets for obsessive compulsive disorder: Evidences from clinical studies

**DOI:** 10.11613/BM.2024.010503

**Published:** 2023-12-15

**Authors:** Aarushi Sultania, Shashank Venkatesan, Dhruv Rishb Batra, Keerthna Rajesh, Rahul Vashishth, Sudesh Ravi, Faraz Ahmad

**Affiliations:** 1 Department of Biotechnology, School of Biosciences and Technology, Vellore Institute of Technology, Vellore, Tamil Nadu, India; 2 Department of Biosciences, School of Biosciences and Technology, Vellore Institute of Technology, Vellore, Tamil Nadu, India

**Keywords:** obsessive compulsive disorder, biomarkers, brain-derived neurotrophic factor, malondialdehyde, dopamine beta-hydrolase

## Abstract

Obsessive compulsive disorder (OCD) is a prevalent behavioral disorder with a complex etiology. However, the underlying pathogenic molecular pathways and the associated risk factors are largely obscure. This has hindered both the identification of relevant prognostic biomarkers and the development of effective treatment strategies. Because of the diverse range of clinical manifestations, not all patients benefit from therapies currently practiced in the clinical setting. Nevertheless, several lines of evidence indicate that neurotrophic, neurotransmitter, and oxidative signaling are involved in the pathophysiology of OCD. Based upon evidences from clinical (and pre-clinical studies), the present review paper sets out to decipher the utilities of three parameters (*i.e.* brain-derived neurotrophic factor; BDNF, noradrenalin-synthesizing enzyme dopamine beta-hydroxylase; DBH; and oxidative damage marker malondialdehyde; MDA) as diagnostic peripheral biomarkers as well as bio-targets for therapeutic strategies. While the data indicates promising results, there is necessitation for future studies to further confirm and establish these. Further, based again on the available clinical data, we investigated the possibilities of exploiting the etiological links between disruptions in the sleep-wake cycle and insulin signaling, and OCD for the identification of potential anti-OCD ameliorative agents with the ability to elicit multimodal effects, including attenuation of the alterations in BDNF, noradrenergic and redox pathways. In this respect, agomelatine and metformin may represent particularly interesting candidates; however, further clinical studies are warranted to establish these as singular or complementary medications in OCD subjects.

## Highlights

The study proposes a set of potential biomarkers for obsessive compulsive disorder

Methods to assess their concentrations in biological samples are critically analyzed

Links between the disorder, diabetes and circadian disruptions are assessed

The implications of biomarkers as therapeutic biotargets are discussed

## Introduction

Obsessive compulsive disorder (OCD) is a neuropsychiatric condition described as an uncontrollable obsession with some specific need, leading to an abnormal compulsive behavior depicted by the subject. The triggers for the obsessions and the compulsive behavior may vary on a patient-to-patient basis. The subjects may elicit a heightened state of irrationality; and while they may be aware of it, they are completely unable to control their tendencies (*1*). With regards to the neuroanatomical and neuropathophysiological aspects, studies have shown that OCD is mainly attributed to the dysfunction of the cortico-striato-thalamo-cortical (CSTC) loop, which is heavily involved in controlling behavioral aspects of motivation, affectivity and sensory-emotional functions, as well as general cognitive functioning. Further, the connectivity between the cerebellum and CSTC may be deterred and spontaneous uncontrolled activation of the circuitry may be observed during OCD-like behaviors (*2*).

Current therapeutic strategies for OCD are majorly restricted to two regimens/agents; cognitive-behavioral therapy using exposure and response prevention (CBT/ERP) and pharmacotherapy based upon serotonergic antidepressants such as selective serotonin reuptake inhibitors (SSRIs) or clomipramine, or a combination of the two. While these have their own set of advantages and disadvantages, data from multiple studies have reported limited responsiveness of OCD patients towards them. A fraction of clinical cases may still remain affected with treatment-refractory OCD after therapeutic interventions (*3*, *4*). Deep brain stimulation (DBS) may be a useful alternative intervention for these subjects who are refractory to other therapeutic regimens (*5*). Needless to say, evaluation of novel and potentially more effective therapeutic strategies is warrantied to address this problem. In this regard, the assessment of endogenous bio-targets involved in the pathogenesis of OCD serves more than one function. First, these may be pertinent targets for novel therapeutic regimens, and second, they may also serve as biomarkers for the diagnosis and prognosis of OCD.

Onset and progression of OCD are thought to have a strong genetic component. Indeed, familial risk of development of OCD has been reported to be as high as 50% in Swedish subjects in a multi-generational study (*6*). Interestingly, maternal genetic effects may contribute significantly to the pathogenesis of OCD (*7*). Surprisingly, not many studies have evaluated the exact identities of the genetic factors (or their single nucleotide polymorphism; SNPs) that may influence disease progression and/or outcome. This might indicate the involvement of a highly complicated network and the interaction between multiple genes, and their associations with environmental risk factors in the development of OCD-like behavior (*8*). Further, animal and clinical studies have stipulated that alterations in neuronal cell survival and synaptic and oxidative signaling potentially contribute heavily to the pathogenesis of OCD. Signaling through brain-derived neurotrophic factor (BDNF) and its tropomyosin receptor kinase B (TrkB) receptor influences multiple aspects of neuronal survival and signaling. Neurotransmitter physiology is another factor that can massively influence brain pathophysiology, including in OCD. Dopaminergic and catecholaminergic signaling in particular are substrates for emotive, social and motivational behavior across multiple brain regions and circuitries. In particular, dopamine beta-hydroxylase (DBH), an enzyme that converts dopamine into norepinephrine may be a critical focal point in OCD pathology as it can potently influence brain circuitry implicated in OCD pathophysiology. Lastly, oxidative stress is an important pathogenic mechanism for OCD. This is hardly surprising given that neurons are particularly vulnerable to oxidative damage and redox alterations are a chief feature of brain pathophysiology in several disorders. Malondialdehyde is a final product of peroxidation-induced damage to polyunsaturated fatty acids (PUFAs), which are abundantly present in the neuronal membranes. It is likely the altered interactions of all three of these factors determine and influence OCD pathology. While this indicates their potential utilities as biomarkers for OCD, it should be noted that currently, there are no diagnostic and prognostic biomarkers for OCD, and diagnostic approaches rely solely on the guidelines provided by the American Psychiatric Association’s Diagnostic and Statistical Manual of Mental Disorders, Fifth Edition (DSM-5) diagnostic criteria for OCD (*9*). The aim of this paper is hence to review the utilities of potentially relevant biomarkers for OCD diagnosis and prognosis in light of the evidence from animal as well as clinical studies. Because of the reasons described above, the three most important potential biomarkers selected are BDNF, DBH, and MDA.

## Identification of relevant biomarkers

Due to onset/progression of a disease, particularly those with a complex and heterogeneous etiology such as OCD, multiple biological parameters are altered (either repressed or induced). Evaluation of the changes in specific biological entities may serve as tools to monitor the presence of the disease. Such bio-targets are called biomarkers (*10*). Identification of specific biomarkers can be used to gauge a variety of factors in consideration for the disorder, such as the approximate time of onset of the disorder, its status and severity, and often the treatment efficacy of a particular therapeutic agent/regimen (*11*). Of note, biomarkers may not be a singular entity, but a combination of parameters can serve the purpose equally well. Further, biomarkers can be any kind of biomolecule, ranging from nucleic acid species to peptides, proteins, lipids and small molecule metabolites.

Identification of relevant diagnostic biomarkers, particularly those present peripherally, in the blood, is a major research aim of neurobiology of disease, particularly for psychiatric disorders such as OCD. In some cases, cerebrospinal fluid (CSF) may also serve as a resource for biomarkers, although its isolation is comparatively more invasive, challenging, and painful. Nevertheless, being derived from the central nervous system (CNS), CSF may represent a more accurate picture with regard to the status and pathology of a neurological disorder (*12*).

## Brain-derived neurotrophic factor

**Figure 1:**
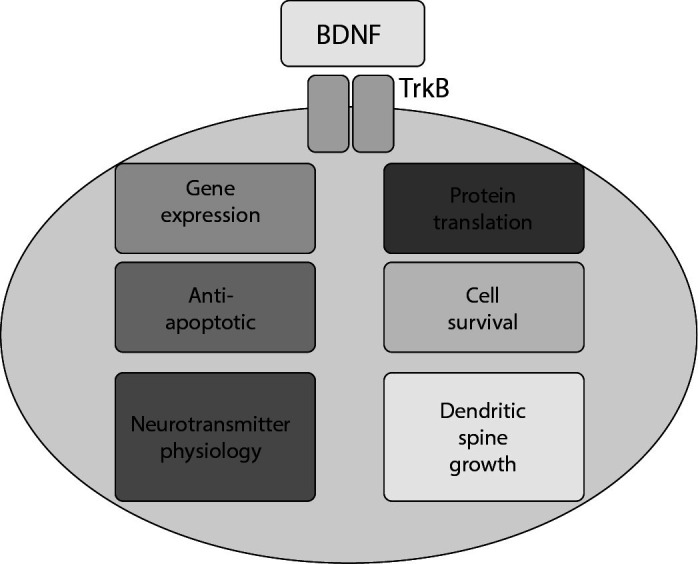
Signaling through brain-derived neurotrophic factor (BDNF) and its tropomyosin receptor kinase B (TrkB). BDNF - TrkB cascade influences a plethora of downstream targets, resulting in multimodal neuromodulatory effects.

Brain-derived neurotrophic factor is a positively charged membrane-binding neurotrophic factor which was the second one to be discovered after nerve growth factor (NGF). Brain-derived neurotrophic factor signaling through its TrkB receptor has multifunctional consequences for neuronal pathophysiology, including for synaptic signaling and plasticity, neuronal survival, astrogliosis and neuroinflammation, and neurogenesis (Figure 1) (*13*). Indeed, BDNF is a potential target for therapies against several neurodevelopmental and neuropsychiatric diseases (*14*). Brain-derived neurotrophic factor is usually secreted by cells in its unprocessed form (pro-BDNF) and is cleaved by various extracellular proteases such as plasmin and matrix metalloproteases (MMPs). Interestingly, pro-BDNF and mature processed BDNF are antagonistic to each other as the former triggers apoptotic pathways while the latter is involved in the activation of cell proliferation and survival pathways (*15*). The cognate membrane receptor for mature BDNF is TrkB, which when triggered in the presence of its ligand, activates several downstream cell survival cascades, including mitogen-activated protein kinase (MAPK)/extracellular signal-regulated kinase (ERK) and phosphoinositide-3-kinase (PI3K) pathways. In humans, the BDNF gene consists of 11 exons, which is two more than in rats and mice; therefore, BDNF is a more complex signaling molecule in humans and animal studies may not necessarily reflect the exact functioning of the protein in the human perspective.

Quantitative assessment of BDNF and pro-BDNF from blood serum is a common strategy employed to diagnose various psychiatric disorders. Enzyme-linked immuno-sorbent assay (ELISA) is the diagnostic standard widely followed under clinical settings, utilizing either anti-BDNF antibodies or TrKB as ligands (*16*). Blood sera and plasma are the most commonly used sources for BDNF-based diagnostics, and whole blood or serum samples are preferred over plasma for ELISA because of stability and sensitivity reasons (*17*). In addition, type of anticoagulant used, clotting duration, and temperature and duration of sample preparation and storage have been shown to influence BDNF quantification in human serum samples (*18*). Mature and pro-forms of BDNF may also be measured in CSF samples collected following lumbar puncture (*19*). Recently, urine has been proposed as a non-invasive source of BDNF measurement, however its utility for the diagnosis of neuronal disorders must be further tested (*20*). Lastly, alternative methods for BDNF measurement, in addition to ELISA may be developed and standardized. An example is immuno-sensing chip-based electrochemical detection of BDNF (*21*).

As discussed, BDNF is the major neurotrophin in the CNS and is involved in multiple neuronal pathways. As such, multiple studies have linked its dysregulated expression to different types of psychiatric disorders (*22*). For these reasons, BDNF may be demarcated as a potent biomarker involved in the pathology of psychiatric disorders such as schizophrenia and OCD (*23*). In particular, the Val-66-Met (rs6265) SNP, located in the terminal exon of the *BDNF* gene is an interesting candidate, and has often been found to be associated with the pathology of neuropsychiatric conditions such as OCD (*24*). The polymorphism has shown to affect trophic signaling of BDNF *via* its TrkB receptor by downregulating the processing of pro-BDNF to mature BDNF (*25*). Interestingly, the Val-66-Met SNP in BDNF may a good predictor of OCD from the early stages of progression in males, however, in women, it is robustly linked to more severe forms of the condition (*25*). Further, meta analyses of multiple studies evaluating the associations of the Val-66-Met variant of BDNF with OCD outcomes have indicated a strong dependence on ethnicity of the subjects, however, there was limited overall linkage between the SNP with OCD pathogenesis (*26*). Similarly, Wang *et al.* have also reported that in their small sample of Chinese Han OCD subjects; there was no association of the Val-66-Met SNP with OCD or generalized anxiety disorder (*27*).

Other SNPs in *BDNF* have also been proposed to be associated with OCD. For example, DNA analyses of blood samples from subjects diagnosed with OCD identified rs2883187 SNP in the *BDNF* gene as a potential risk factor for the development of OCD (*28*). Since this SNP is in the intronic region of the *BDNF* gene, it probably influences the expression and/or mRNA splicing, rather than impacting the morpho-functional aspects of BDNF *per se* (*29*). Interestingly, methylation and hydroxymethylation status of the promoter regions of *BDNF* from mononuclear cells in peripheral blood has also been proposed as an indicator of OCD pathology (*30*). Of note, saliva samples have also been demonstrated to be a viable source of epigenetic analyses of *BDNF* gene in OCD subjects in a recent study (*31*).

Absolute BDNF concentrations in peripheral blood samples may also be a potential biomarker for OCD, both in clinical cases under drug-treatment, and with no medication (*32*, *33*). A meta-analysis revealed that OCD subjects had significantly downregulated BDNF concentrations in their blood, compared to controls. Moreover, BDNF may also serve as a prognostic factor as OCD subjects under medication elicit increased blood BDNF concentrations when compared to subjects without any drug treatment (*34*). It should be noted here that quantification of peripheral BDNF concentrations in blood/serum samples provide a good representation of its expression in the brain tissues, as evidenced from chemical models of OCD induced by dopaminergic antagonist quinpirole in rats (*35*). Moreover, serum BDNF concentrations may also be a pertinent biomarker for OCD in children (*36*). In 7-17 years old children diagnosed with OCD, serum BDNF concentrations were shown to be elevated in comparison to healthy aged-matched controls, possibly as part of the former’s adaptive compensatory mechanisms to counteract aberrant activation of the hypothalamic-pituitary-adrenal (HPA) axis (*37*).

Interestingly, post-translational processing of BDNF also seems to be impacted during OCD pathogenesis. Thus, the ratio of plasma concentrations of processed mature BDNF to unprocessed pro-BDNF has been found to be significantly repressed in clinical cases of OCD (*38*). Post-transcriptional mechanisms may also be at play in determining deficits BDNF expression and activity in OCD. Indeed, a risk allele of micro-RNA, miR-30a-5p has been identified in clinical cases of OCD; interestingly it can differentiate between early and late onset OCD (*39*). Given that miR-30a-5p is a potent mediator of BDNF signaling and acts by downregulating its expression, it will be interesting to identify the underlying mechanisms of the linkage between the miR-30a-5p/BDNF axis and OCD.

## Malondialdehyde

Oxidative stress and damage to biomolecules (lipids, nucleic acid species and proteins) is an integral aspect of neurobiology of mood and behavioral disorders, resulting in disintegrity of the blood brain barrier, neuroinflammation and deficits in neurotransmitter signaling, among other consequences (*40*). Unsurprisingly, alterations in redox signaling have been proposed to contribute significantly to the pathogenesis of OCD, to the extent that the well-known antioxidant, N-acetyl cysteine (NAC) has been proposed as a therapeutic agent against OCD (*41*, *42*). Indeed, clinical data suggests that biochemical parameters of redox status are excessively altered in serum samples of OCD subjects. Thus, they elicited upregulated measures of total oxidant status (TOS) and oxidative stress index (OSI), concomitantly with downregulated levels of total antioxidant status (TAS) in comparison to healthy aged-matched controls (*43*).

**Figure 2:**
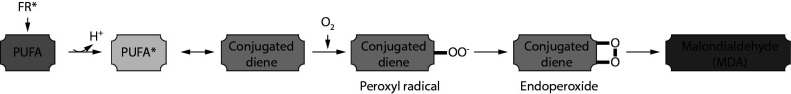
Malondialdehyde (MDA) formation *via* peroxidation of polyunsaturated fatty acids (PUFAs). The figure represents the biochemical steps involved in the formation of MDA from PUFAs initiated by free radical attack (FR*).

An important marker for oxidative stress is MDA, a three-carbon molecule formed by the lipid peroxidation of PUFAs (*e.g.* arachidonic acid). Due to the action of free radical species, PUFAs are broken down into MDA, which is a stable signaling molecule (Figure 2). The simplest method for the estimation of MDA in whole blood, serum or plasma, CSF, saliva, and urinary samples obtained from human subjects is *via* spectroscopic analysis, although fluorimetric, high performance liquid chromatography (HPLC)-based chromatographic and liquid chromatography - mass spectrometry (LC-MS) -based mass spectrophotometric assays are also commonly used (*44*). The biological sample must be pre-treated with trichloroacetic acid to precipitate all proteins. Being a prototype thiobarbituric acid (TBA) reactive substance (TBARS), detection of MDA is based upon its ability to react with TBA to form a chromogenic product that can be quantified on a spectrophotometer at 530 nm. A major drawback of this method is its non-specificity, as other aldehydes present in the sample can also react with TBA and interfere with the analyses (*45*). The assay can thus be coupled with HPLC or LC-MS for effectiveness, particularly in sparse samples. Further, derivatization of MDA with 2,4-dinitrophenylhydrazine (DNPH) prior to separation on HPLC columns may ensure specificity of the reaction (*46*). The thiobarbituric acid assay can also be used for quantification of MDA in CSF, although the pre-treatment steps differ and may include addition of surfactants and acids to the supernatant obtained upon centrifugation (*47*).

Increased MDA concentrations in peripheral blood samples has been proposed as a biochemical biomarker for the diagnosis and evaluation of the severity of the symptoms of OCD in several independent studies (*48*-*52*). A recent meta-analysis indicated that serum/plasma concentrations of MDA are positively correlated OCD pathology in human subjects (*53*). Such systemic redox imbalances have also been proposed by another independent meta-analytical study, wherein the authors reported significant positive association of oxidative damage markers such as 8-hydroxydeoxyguanosine (8-OHdG) and MDA, in addition to a negative correlation of endogenous antioxidant systems (*e.g.* glutathione peroxidase; GPx and superoxide dismutase; SOD) with OCD pathology in the blood samples of diseased human subjects (*54*).

## Dopamine beta-hydroxylase

**Figure 3:**
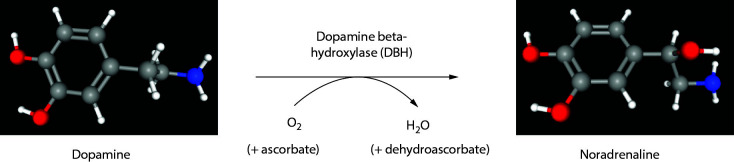
Reaction catalyzed by dopamine beta-hydroxylase (DBH). Dopamine is converted to noradrenalin (norepinephrine) in the presence of molecular oxygen and ascorbate in a reaction catalyzed by DBH. Water and dehydroascorbate are released as by-products.

Synapses are the primary pathways of interneuronal communication and the substrates for high-order behaviors such as emotive and social functions. Neurotransmitters; dopamine, serotonin, noradrenaline/norepinephrine (NE) and glutamate hence play important roles in the pathophysiology of neuropsychiatric disorders such as OCD (*55*). In particular, deficits in dopaminergic and serotonergic signaling have been proposed as critical pathogenic mechanisms for the development and progression of OCD since some time now (*56*). Noradrenergic signaling is another neurotransmitter pathway which is known to be involved in the pathophysiology of OCD (*57*, *58*). In concurrence, increased concentrations of norepinephrine have been observed in the CSF of human subjects with OCD, and mouse models have shown the requirement of altered noradrenergic signaling in OCD-like behavior (*59*, *60*). Dopamine beta-hydroxylase is an enzyme which is crucial for the production of NE from dopamine (Figure 3), and hence is a critical point of regulation in the biosynthesis of catecholamines. Animal experiments with DBH knockout mice suggest its crucial involvement in OCD-related excessive grooming, marble burying and nest-shredding behaviors (*60*, *61*). Importantly, presence of DBH in systemic circulation may allow it to serve as potential peripheral biomarker for noradrenergic signaling and hence, neuronal disorders such as OCD (*62*). Indeed, recent research has demonstrated the presence of significantly elevated concentrations of DBH enzyme in the serum of drug-naïve OCD subjects (*63*). In addition, polymorphisms of DBH may also be a target for biomarker identification against behavioral disorders, including OCD (*62*, *64*, *65*). Nevertheless, more studies with greater sample sizes are needed to affirm the linkages between the concentrations of DBH (and its polymorphisms) and OCD pathophysiology. This may be relevant considering that plasma DBH concentrations are not linked to the pathology of Tourette’s syndrome (TS), which closely resembles OCD in the etiology and morbidity (*66*). Hence, peripheral DBH concentrations may represent a specific biomarker for OCD in this respect.

Dopamine beta-hydroxylase concentrations exhibit significant variations among individuals, influenced by a combination of genetic factors, age, and health conditions. Genetic polymorphisms, for instance, can exert a profound impact on DBH activity (*62*). To measure DBH activities in CSF samples, two methods have been employed; a dual-wavelength spectrophotometric method, and HPLC with fluorescence detection. Both methods rely on tyramine, a structural analogue of dopamine, as the substrate, ensuring optimal conditions for DBH activity assessment. The dual-wavelength spectrophotometric method measures p-hydroxybenzaldehyde formation, while HPLC is employed to quantitate the conversion of tyramine to octopamine (*67*, *68*). In addition to these direct measures, DBH metabolites like homovanillic acid (HVA) and vanillylmandelic acid (VMA) can also be assessed. Thus, gas chromatography - mass spectrometry (GC-MS)-based quantification of the HVA/VMA ratio in urine samples has been used as a measure of epinephrine/norepinephrine imbalance and altered dopamine production, which could be related to DBH (*69*). It should be noted that assaying DBH activity presents significant challenges. The various techniques used, such as spectrophotometry, radioenzymatic assays, and immunoassays may yield slightly different results, thereby making standardization a problem. Ensuring that the assays specifically measure DBH activity without interference from other enzymes or substances can also be challenging. Furthermore, DBH activity in CSF has been found to be less stable than in serum, possibly due to its low concentration in CSF (*62*). Hence, it is obvious that improvements in detection of DBH activity in human blood and CSF samples are warrantied.

## Potential biomarkers as therapeutic targets for OCD

A comprehensive understanding of the underlying molecular mechanisms is required for linking the potential biomarkers discussed above with OCD pathophysiology. In addition, they may serve as bio-targets for therapeutic strategies. It should be noted here that the three biomarkers discussed here are closely inter-related. Thus, oxidative stress can be induced by enhanced secretion of noradrenaline, leading to release of free radicals from the mitochondria. The free radicals directly affect BDNF-TrkB signaling. Hence, a reciprocal correlation can be drawn between concentrations of noradrenalin (and reactive oxygen species (ROS)) with BDNF; as DBH activity increases, noradrenalin increases and causes elevation in ROS concentrations and depletion in BDNF concentrations.

Obsessive compulsive disorder research has advanced significantly during the past decades. The most effective clinical care of OCD involves early intervention and relapse prevention techniques. Adults with OCD may then be treated with selective serotonin reuptake inhibitors (SSRIs) or cognitive behavioral therapy (CBT). A combinatorial approach involving both may also be employed as the first-line of therapy (*70*). However, data indicates that a percentage of OCD subjects may be resistant to such first-line treatments. In such cases, antipsychotics, such as aripiprazole and risperidone may be included in the treatment regimen (*71*). Data from both animal and human studies indicate that risperidone mitigates the downregulation of BDNF and consequently has beneficial effects on neuroinflammatory and redox signaling cascades (*72*-*76*).

Deep brain stimulation (DBS) is a novel therapeutic regimen, originally developed for the treatment of movement disorders. Although this needs to be confirmed and established, recent studies indicate that DBS may represent a therapeutically relevant option against neuropsychiatric conditions, including OCD (*77*, *78*). Moreover, multiple preclinical and clinical studies have indicated that DBS may rescue the detrimental deficits in BDNF signaling, in addition to mitigation of pathological aberrations in noradrenergic (*79*-*85*).

Altered melatonin signaling and disrupted circadian rhythm have been found to be co-morbid with OCD and melatonin concentrations are often found to be downregulated in serum samples from clinical cases (*86*-*89*). Melatonergic agonist, agomelatine has been proposed as an ameliorative agent against OCD in many clinical studies, particularly against subjects who are unresponsive to conventional first-line and second-line therapeutic regimens (reviewed in (*90*)). Apart from its beneficial effects on circadian rhythm, it also improves serotonergic and noradrenergic signaling (*91*, *92*). Not surprisingly, agomelatine therapy in human subjects may result in amelioration of OCD-linked symptoms by 90% (*93*). In addition, clinical studies have also indicated significant synergistic beneficial effects of agomelatine supplementation on BDNF levels in patients (*94*-*96*). Further, preclinical data suggests that agomelatine may also target oxidative pathways and repress the formation of MDA; nevertheless, studies are warranted to ascertain this in humans (*97*-*99*).

Recent research suggest that chronic metabolic conditions, such as diabetes contribute massively to the increased risk of neurological disorders, including mood disorders such as OCD (*100*). A bidirectional relationship has been proposed between altered insulin signaling and OCD pathogenesis (*101*). Obsessive compulsive disorder and insulin-linked metabolic conditions may share a common etiology with regard to genetic risk factors (*102*). In concurrence, clinical studies in OCD subjects have reported altered plasma concentrations of insulin and insulin-like growth factor-1 (IGF-1) (*103*, *104*). Conversely, studies have reported psychological vulnerabilities and a high prevalence of OCD-like symptoms in subjects with diabetes mellitus (*105*, *106*). Animal studies also support such bidirectional interaction between insulin signaling and OCD (*107*, *108*). Given the massive implications of insulin signaling in neuronal pathophysiology, the deficits in insulin and IGF-1 signaling may be hypothesized to affect OCD pathophysiology in a multimodal manner (*109*). For example, dendritic spine dynamics represent a crucial manner *via* which insulin signaling regulates neurotransmitter signaling through the glutamatergic, serotonergic, noradrenergic, and dopaminergic systems (*110*). Nevertheless, the exact identities of the molecular players and mechanisms are yet to be discerned. This is important since there is some evidence that drugs against OCD (*e.g.* risperidone) may result in severe problems in insulin signaling, particularly if the subject has a family history of diabetes mellitus (*111*). Importantly, data suggesting the possible involvement of insulin signaling in OCD pathology opens up the possibility of developing novel therapeutic agents and strategies. For example, genistein, a potential anti-diabetic drug may be useful in ameliorating OCD-like behavior in animal models (*108*, *112*). Similarly, metformin, used for the treatment of diabetes has been hypothesized to elicit potentially beneficial effects against OCD (*101*, *113*). Indeed, a recently published study reported the ameliorative effects of metformin in reversing metabolic and body weight alterations induced by antipsychotic treatment regimens in a Canadian subject (*114*).

Lastly, detrimental alterations in glutamatergic signaling may play notable roles in the pathophysiology of OCD. Research exploring these links has shown that patients display a peculiar hyperactivation of glutamatergic signaling in the cortico-striatal-thalamo-cortical circuit. Psychological and cognitive dysfunctions elicited by OCD patients may be associated with the changes in the concentrations of glutamate and its signaling functions, particularly *via* the N-methyl-D-aspartate (NMDA) receptors (*117*). However, establishing this link precisely has been an arduous task since glutamatergic neurons are common to all major regions of the brain. Further, glutamatergic agents like memantine, ketamine, and rapastinel, have been evaluated for alleviation of OCD symptoms with reasonable successes (*118*). Another potential pathogenic pathway of OCD development is centered on hyperactivation of neuroinflammatory system (*119*). Studies have revealed concurrent incidences of neuroinflammatory pathologies like autoimmune dysfunctions of lupus and multiple sclerosis in OCD patients. Additionally, altered expression of inflammatory mediators has been observed during OCD pathogenesis, leading to the proposition that immunomodulatory agents may be employed for augmenting therapeutic strategies against OCD (*120*). While the pathogenic links between aberrant activation of glutamatergic and inflammatory signaling, and OCD development have been proposed, further research is needed to confirm and establish them as diagnostic and therapeutic targets.

## Conclusions and future directions

Obsessive compulsive disorder etiology is complex and the underlying pathogenic pathways and risk factors are largely undiscerned. Since OCD has a diverse range of clinical manifestations, not all patients benefit from therapies currently practiced in the clinical setting. Nevertheless, several lines of evidences indicate that neurotrophic, neurotransmitter, and oxidative signaling cascades are involved in the pathophysiology of OCD. The present review paper sets out to decipher the utilities of three main representative parameters for these cascades as potential biomarkers (and therapeutic targets). These include BDNF which is a neurotrophin with multimodal effects on neuronal survival and signaling, DBH which is involved in the conversion of dopamine to norepinephrine, and therefore critically influences the synaptic signaling, and MDA which is a biomolecular indicator of oxidative damage to lipids. It should be noted here that only a thorough understanding of the interrelations of these three factors and their simultaneous and cumulative changes in OCD pathology may present a clear indication of the disease status. Further, we have also discussed co-morbidities of chronic sleep disturbances and diabetes as these can further provide pertinent information about the status and severity of the neurological conditions in OCD, and may serve to identify potent therapeutic targets. In this regard, agomelatine and metformin may represent particularly interesting candidates; however, further clinical studies are warranted to establish these as singular or complementary medications in OCD subjects.

Another aspect for future research is the requirement to identify the exact molecular players and pathways involved in OCD pathogeneses, which will be beneficial in confirming the suitability of the three candidates as biomarkers and therapeutic targets. While modelling behavioral conditions with a complicated etiology, such as OCD is challenging in animals, it represents one of the major advantageous strategies for the study of the pathogenic mechanisms of, and evaluation of interventions against these diseases (*115*). Modelling of neurocircuitry may represent another relevant strategy to understand the neurological and cognitive mechanisms underlying OCD, although there are several limitations associated with these (*116*). Perhaps, a combinatorial approach involving animal, clinical, and *in silico* studies is needed for understanding and treating OCD, and confirming and establishing the utility of BDNF, MDA and DBH as relevant biomarkers and targets in OCD pathophysiology.

## Data Availability

No data was generated during this study.
